# Coherent neural oscillations inform early stroke motor recovery

**DOI:** 10.1002/hbm.25643

**Published:** 2021-08-26

**Authors:** Jessica M. Cassidy, Anirudh Wodeyar, Ramesh Srinivasan, Steven C. Cramer

**Affiliations:** ^1^ Department of Allied Health Sciences University of North Carolina Chapel Hill North Carolina USA; ^2^ Department of Cognitive Sciences University of California Irvine Irvine California USA; ^3^ Department of Biomedical Engineering University of California Irvine Irvine California USA; ^4^ Department of Neurology University of California, Los Angeles Los Angeles California USA; ^5^ California Rehabilitation Institute Los Angeles California USA

**Keywords:** biomarker, electroencephalography, motor cortex, stroke

## Abstract

Neural oscillations may contain important information pertaining to stroke rehabilitation. This study examined the predictive performance of electroencephalography‐derived neural oscillations following stroke using a data‐driven approach. Individuals with stroke admitted to an inpatient rehabilitation facility completed a resting‐state electroencephalography recording and structural neuroimaging around the time of admission and motor testing at admission and discharge. Using a lasso regression model with cross‐validation, we determined the extent of motor recovery (admission to discharge change in Functional Independence Measurement motor subscale score) prediction from electroencephalography, baseline motor status, and corticospinal tract injury. In 27 participants, coherence in a 1–30 Hz band between leads overlying ipsilesional primary motor cortex and 16 leads over bilateral hemispheres predicted 61.8% of the variance in motor recovery. High beta (20–30 Hz) and alpha (8–12 Hz) frequencies contributed most to the model demonstrating both positive and negative associations with motor recovery, including high beta leads in supplementary motor areas and ipsilesional ventral premotor and parietal regions and alpha leads overlying contralesional temporal–parietal and ipsilesional parietal regions. Electroencephalography power, baseline motor status, and corticospinal tract injury did not significantly predict motor recovery during hospitalization (*R* ^2^ = 0–6.2%). Findings underscore the relevance of oscillatory synchronization in early stroke rehabilitation while highlighting contributions from beta and alpha frequency bands and frontal, parietal, and temporal–parietal regions overlooked by traditional hypothesis‐driven prediction models.

## Graphical abstract

This study examined the predictive performance of electroencephalography‐derived neural oscillations following stroke using a data‐driven approach. In 27 participants, EEG coherence in a 1–30 Hz band between leads overlying ipsilesional primary motor cortex and 16 leads over bilateral hemispheres predicted 61.8% of the variance in motor recovery.

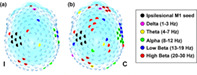

## 1 INTRODUCTION

Stroke is the leading neurological cause of disability in the United States (Feigin et al., 2021) with motor deficits constituting a significant source of poststroke burden. A multitude of restorative therapies such as noninvasive brain stimulation, stem cells, biologicals, robotics, and activity‐ and cognitive‐based therapies are emerging; however, the heterogeneity of stroke complicates the assessment of their efficacy. Biomarkers, particularly those developed from neuroimaging, can address this heterogeneity to improve understanding and enhance the accuracy of predicting clinical outcomes and treatment responses. Measurements derived from structural neuroimaging are well established in the literature and deemed ready for clinical research implementation (Boyd et al., 2017). In contrast, the utility of functional neuroimaging measurements from functional magnetic resonance imaging (fMRI), electroencephalography (EEG), and magnetoencephalography (MEG) requires further investigation as a developmental priority (Boyd et al., 2017). Application of these neuroimaging modalities in stroke to examine functional neuronal connections during rest align with the emerging view of stroke as a disease of “circuitopathies (Meder & Siebner, 2018)”, whereby abnormal patterns of connectivity contribute to behavioral deficits.

EEG is an appealing neuroimaging modality for biomarker development given its portability, safety, accessibility across participants and clinical sites, and low cost in comparison to fMRI and MEG. EEG directly captures electrical potentials at the scalp surface that represents synchronized synaptic activity generated predominantly from neural tissue comprised of hundreds of millions of neurons (Nunez, Nunez, & Srinivasan, 2019) underlying the EEG electrode. The oscillations recorded from EEG reflect brain function including the control and timing of neuronal firing and information transfer across brain regions (da Silva, 1991) in addition to higher‐order processes related to cognitive and motor function (Günseli et al., 2019; Jensen, Kaiser, & Lachaux, 2007; Knyazev, 2012; Pfurtscheller, Brunner, Schlögl, & Da Silva, 2006; Tomassini, Ambrogioni, Medendorp, & Maris, 2017). Resting‐state EEG has provided valuable insights in stroke by highlighting the relevance of connectivity‐related measurements such as coherence (Cassidy et al., 2020; Dubovik et al., 2012; Wu et al., 2015). As a measure signifying the degree of consistency of amplitude and phase differences between two signals in a specific frequency band across time, many consider coherence as a surrogate measure of communication or functional connectivity between spatially distinct brain regions (Srinivasan, Winter, Ding, & Nunez, 2007). Despite physiological differences between EEG and fMRI blood‐oxygen‐level‐dependent signals, there is a consistency of findings between the two indicating disordered sensorimotor network functional connectivity following stroke that may normalize over time in proportion to motor recovery (Nicolo et al., 2015; Park et al., 2011). Resting‐state EEG coherence measurements may therefore serve as a potential stroke biomarker to characterize and predict motor status and outcomes (Saes, Meskers, Daffertshofer, van Wegen, & Kwakkel, 2020; Wu et al., 2015) while illustrating interactions across various neural networks beneficial to motor recovery.

Two competing lines of scientific inquiry influence the development of stroke prediction models. Traditional hypothesis‐driven models, formulated from prior knowledge and experimentation, provide plausible biological explanation. Whereas data‐driven approaches, for example, machine learning, do not rely on these factors, and may be more suitable for large neuroimaging datasets typified by multiple regions of interest, network nodes, parcellations, EEG/MEG leads, and frequency bands. While machine learning only prioritizes prediction accuracy, there exist several statistical regression approaches meant to enhance both prediction accuracy and interpretation while remaining data‐driven. Statistical regularized regression approaches are particularly useful when there exist a high number of predictors and the presence of multicollinearity. Regularized regression approaches, including ridge (Hoerl & Kennard, 1970), elastic net (Zou & Hastie, 2005), and least absolute shrinkage selector operator (lasso) (Tibshirani, 1996) regression approaches, address these shortcomings by penalizing regression coefficients to produce simpler (more generalizable) models without overfitting the data. Penalizations occurring in lasso regression may result in the elimination of variables from the model. In contrast, variables remain in ridge regression models as their values may approach but never reach zero following the application of an L_2_ penalty. Elastic net combines characteristics of lasso and ridge regression and their respective L_1_ and L_2_ penalties (Zou & Hastie, 2005). Collectively, these regularization and shrinkage methods for regression provide a more robust means of estimating outcomes in stroke.

A thorough review of penalized least squares regression approaches in neuroimaging is available elsewhere (Bunea et al., 2011), but several pertinent examples of lasso application exist. Work by Kohannim et al. (2012) employed lasso regression to assess gene effects in genome‐wide association studies of brain MRI images from over 700 subjects participating in the Alzheimer's disease neuroimaging initiative (Kohannim et al., 2012). Recent work by Cole implemented lasso regression when combining several neuroimaging modalities including T1, T2‐FLAIR, T2*‐weighted and diffusion MRI along with task and resting‐state fMRI for brain‐age prediction (Cole, 2020). Quinlan et al. (2015) confirmed a prior multivariate regression model using lasso regression that found CST injury and resting‐state fMRI connectivity between bilateral motor cortices best predicted treatment gains from 3 weeks of robotic upper extremity therapy (Quinlan et al., 2015). Notably, investigators inspected over 30 candidate predictors across subject demographics/medical history, cognition and mood, impairment, genetics, brain injury, cortical function, and cortical connectivity categories (Quinlan et al., 2015). Finally, Erani et al. (2020) identified a subset of electrode pairs that best diagnosed acute stroke in the emergency department using lasso regression (Erani et al., 2020).

Our prior EEG work in subacute stroke found (1) negative associations between beta power in leads overlying ipsilesional sensorimotor and contralesional parietal cortices with motor impairment and global stroke severity status (Wu et al., 2016), (2) positive associations between delta power in ipsilesional sensorimotor and contralesional frontoparietal cortices (Wu et al., 2016) with motor impairment and global stroke severity status, and (3) reductions in delta coherence between bilateral primary motor cortices paralleling motor recovery (Cassidy et al., 2020). Informed by these findings, traditional hypothesis‐driven prediction models for early stroke motor recovery would thus contain contributions from both high‐ and low‐frequency bands in leads overlying bilateral primary and secondary motor regions in early stroke motor recovery.

This current study, however, took a more data‐driven approach for motor recovery prediction using lasso regression to determine the predictive performance of neural oscillations acquired from a dense‐array (256 leads) EEG system during early stroke rehabilitation. Broadly, we hypothesized that applying lasso regression would identify a subset of leads and frequency bands that significantly predict motor recovery during inpatient rehabilitation after stroke. Based on past work underscoring the significance of resting‐state oscillations in alpha (Dubovik et al., 2013), delta (Cassidy et al., 2020), theta (Saes et al., 2020), and beta (Wu et al., 2015) frequency bands along with functional connections with ipsilesional primary motor cortex (iM1) spanning both ipsi‐ and contralesional hemispheres, lasso regression is an appropriate strategy for the prediction of motor recovery during poststroke hospitalization that may reveal additional findings not otherwise apparent through mainly traditional hypothesis‐driven approaches.

## 2 METHODS

### 2.1 Participants

Individuals with ischemic stroke or intracerebral hemorrhage aged 18 years or older were recruited from the inpatient rehabilitation facility at the University of California, Irvine Medical Center. Exclusion criteria included substantial communication deficits, contraindication to MRI, and a history of cranial surgery that might introduce a breach rhythm in the EEG signal. Participants completed motor testing around IRF admission and discharge that entailed the Upper Extremity Fugl‐Meyer (UEFM) and Functional Independence Measurement motor subscale (FIM‐motor). Participants also completed a 3‐min resting state EEG recording and a structural magnetic resonance imaging (MRI) scan around the time of IRF admission. This study received approval from the University of California, Irvine Institutional Review Board. All participants provided written informed consent.

### 2.2 Procedures

#### 2.2.1 Electroencephalography acquisition and preprocessing

A 3‐min resting‐state EEG recording was obtained from each awake participant with a dense‐array 256‐lead Hydrocel net (Electrical Geodesics Inc., Eugene, OR). As in our prior studies (Cassidy et al., 2020; Wu et al., 2015), EEG data were sampled at 1,000 Hz using a high input impedance Net Amp 300 amplifier and Net Station 4.5.3 software (Electrical Geodesics Inc.). Raw and unfiltered EEG data were imported to Matlab (Mathworks, Natick, MA) for offline preprocessing that involved the following steps: (1) re‐referencing to the average signal across all leads after the removal of 64 leads from cheek and neck regions, (2) 50 Hz low‐pass filtering, (3) segmenting the data into 1‐s nonoverlapping epochs with detrending, and (4) muscle artifact removal during visual inspection. Ocular and cardiac artifacts were removed using an Infomax independent components analysis (ICA) in EEGLAB (Delorme & Makeig, 2004) prior to an additional round of visual inspection with data transformed to electrode space to assess ICA accuracy.

#### 2.2.2 Electroencephalography measurements

Spectral analysis of the data using a discrete Fast Fourier transform allowed for the computation of power and coherence at all 192 leads from 1 to 30 Hz. Measures of relative power for each electrode were obtained by dividing power in a specific frequency band (delta, 1–3 Hz; theta, 3–7 Hz; alpha, 8–12 Hz; low beta, 13–19 Hz; high beta, 20–30 Hz) by the total power summed over the entire 1–30 Hz range. Stroke lesions located in the right hemisphere were flipped to the left hemisphere so that the left hemisphere constituted the ipsilesional hemisphere for analysis of all participants. The primary seed region for coherence measurements encompassed a set of predefined electrodes (C3 and the surrounding six leads) overlying iM1. Coherence measurements in subsequent analyses were calculated as the squared correlation coefficient with values ranging from 0 to 1. Values approaching 1 represent consistency in phase and amplitude ratios.

#### 2.2.3 Magnetic resonance imaging

Structural imaging including a high resolution T1‐weighted scan that included a three‐dimensional magnetization‐prepared rapid gradient echo sequence (repetition time (TR) = 8.1 ms, echo time (TE) = 3.7 ms, 150 slices, voxel size 1 × 1 × 1 mm^3^) and a T2‐weighted fluid‐attenuated inversion recovery (FLAIR) scan (TR = 9,000 ms, TE = 120 ms, 33 slices, voxel size 0.58 x 0.58 x 5 mm^3^) were acquired on a Philips Achieva 3‐Tesla scanner (Best, the Netherlands).

#### 2.2.4 Lesion masks and corticospinal tract injury

Lesion masks for each participant were hand‐drawn on T1‐weighted MRI scans as further informed by the T2‐FLAIR scan using methods with established reliability and validity as previously described (Burke et al., 2014). Masks were binarized and transformed to Montreal Neurological Institute (MNI) space. CST injury was assessed as the percentage overlap between lesion masks and a CST template generated from 28 healthy individuals as part of the Johns Hopkins University tractography atlas (Hua et al., 2008).

### 2.3 Statistical analysis

Statistical tests were performed using Matlab and JMP Pro 14.0.0 (SAS Inc., Cary, NC). Prediction of motor recovery (admission to discharge FIM‐motor change) using EEG measurements was done using the Matlab function *lassoglm* which includes hyperparameter optimization using leave‐one‐out cross‐validation.

We adjusted the degree to which the lasso penalization term (L_1_ parameter) was weighted relative to the ridge penalization term (L_2_ parameter). Adjusting the L_1_ parameter from 1 to 0.80, indicating a weight of 0.2/2 for the L_2_ parameter, implemented both lasso and ridge regression properties otherwise known as elastic net regularization. We performed elastic net regression for the purpose of interpretation rather than prediction given the likelihood of the identification of additional leads. Because of issues of multicollinearity with EEG, elastic net regression allows for the inclusion of correlated predictors while continuing to negate uninformative leads. Since elastic net regularization includes the L_1_ parameter, it is a more appropriate strategy to employ for the purpose of meaningful interpretation as compared to ridge regression where coefficients are only minimized and not set to zero.

Motor recovery prediction using CST injury and baseline motor impairment status were assessed with general linear regression models. Normal distribution of the dependent variable and resulting model errors was confirmed with the Shapiro–Wilk test.

## 3 RESULTS

### 3.1 Participants

**FIGURE 1:**
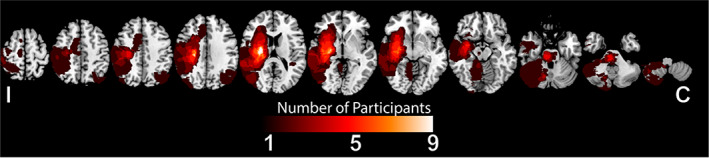
Participant stroke masks on T1‐weighted images. Lighter colors indicate greater frequency of injury across participants. C, contralesional hemisphere; I, ipsilesional hemisphere. Two participants sustained bilateral injury

A cohort of 27 individuals with predominantly mild–moderate motor impairment (UEFM average score = 43.7 ± 19.8 points, 66 point maximum, higher is better) receiving inpatient rehabilitation participated. Two participants sustained stroke‐related damage to bilateral hemispheres. In these cases, the ipsilesional hemisphere was classified as the hemisphere opposite to the upper‐extremity depicting more profound motor deficit. Median time from IRF admission to study enrollment was 5 days [IQR =  2‐9]. Over the course of their hospitalization (average IRF length of stay = 20.9 ± 21 days), participants made substantial motor recovery as evidenced by FIM‐motor change from IRF admission to discharge (33.8 ± 14.1 points, score ranges from 13 to 91, with higher values representing better motor function). Table 1 reports additional study cohort characteristics. Figure 1 illustrates stroke‐related injury across participants.

**TABLE 1:** Participant characteristics (*N* = 27)

Measures	Value
Age (years)	58.3 ± 14.6
Sex (male/female)	20/7
Time poststroke (days)	12 [8–17]
Stroke type (ischemic/hemorrhagic)	21/6
Lesion side (right/left)	16/11
Lesion volume (cc)	18.7 ± 25.1
Percent CST injury	45.2 ± 35.9
NIH stroke scale (0–42 points)	3 [2–6]
Admission FIM‐motor score (13–91 points)	37.9 ± 11.9
Discharge FIM‐motor score (13–91 points)	71.7 ± 13.1
FIM‐motor change	33.8 ± 14.1
Admission UEFM (0–66 points)	43.7 ± 19.8

*Note*: Values presented as mean ± SD or median [interquartile range].

Abbreviations: FIM‐motor, Functional Independence Measurement motor subscale; UEFM, Upper Extremity Fugl‐Meyer.

### 3.2 EEG measurements and motor recovery

**FIGURE 2:**
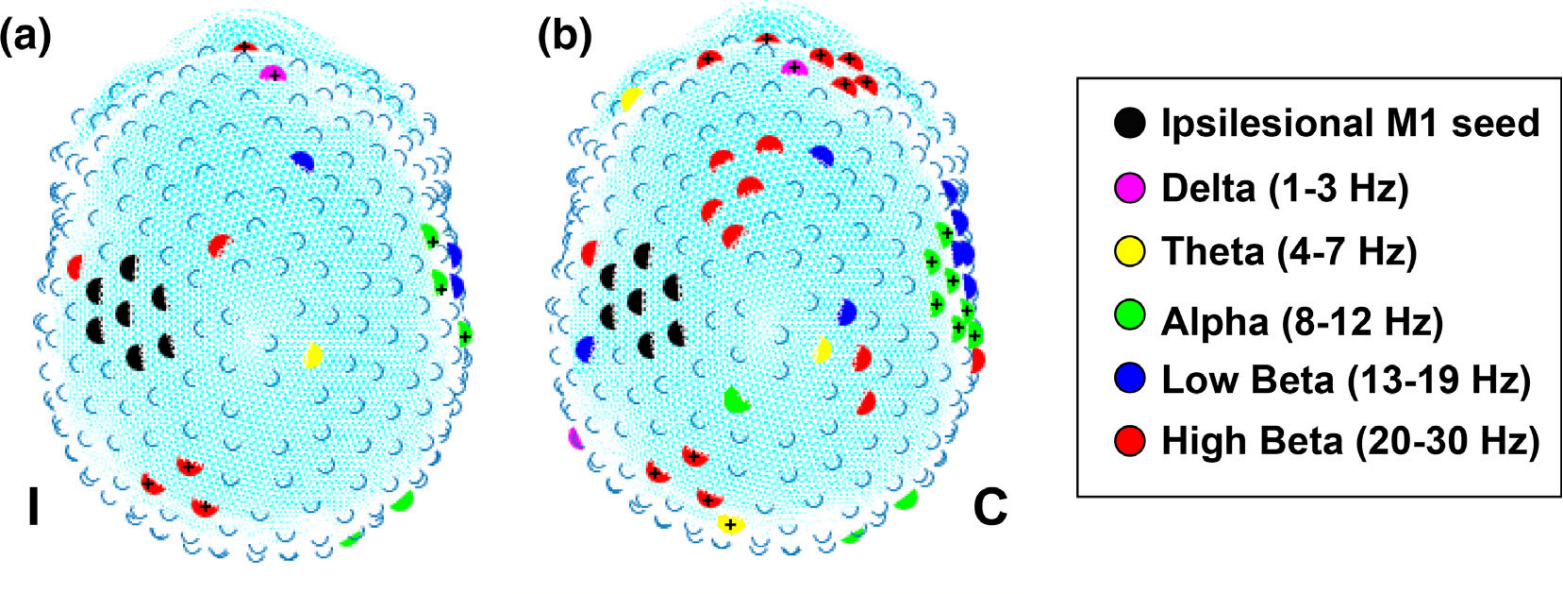
Across a 1–30 Hz frequency band, 16 electrodes identified through lasso regression explained 61.8% of FIM‐motor change from inpatient rehabilitation facility admission to discharge (a). An elastic net model identified 49 electrodes that explained 46.9% of the variance in FIM‐motor change (b). + symbol corresponds to a positive beta coefficient value in the model, otherwise the coefficient was negative. In instances where the model identified multiple frequency bands for a given electrode, the figure displays the frequency with the greatest absolute value of the beta coefficient for that electrode

Across a 1–30 Hz band, EEG coherence between leads overlying iM1 and 16 leads located in both ipsi‐ and contralesional hemispheres explained 61.8% of the variance in motor recovery (Figure 2a). To further examine the spatial distribution of leads and corresponding frequencies for the purposes of interpretation outside of predictive ability, we adjusted the degree to which the lasso penalization term was weighted relative to the ridge penalization term to reflect elastic net regularization. The elastic net model resulted in an expanded collection of 49 leads coherent with leads overlying iM1, explaining 46.9% of the variance in motor recovery (Figure 2b). Model predictor (EEG leads) information from both lasso and elastic net models are summarized in Table 2. Interpreting parameter values across frequency bands, we find that in the low beta band, all regression coefficients are negative indicating that reduced coherence in low beta implied increased motor recovery, given all other variables remain constant. In contrast, in the delta band, the majority of regression coefficients are positive, suggesting that increases in delta coherence with iM1 result in increased motor recovery scores. In alpha, theta, and high beta bands, however, there is a range of parameter weights spanning positive and negative values suggesting that there is area specificity to how changes in coherence are related to motor recovery in these bands. No linear combination of the relative power values provided useful predictive power for motor recovery (*R* ^2^ = 0).

**TABLE 2:** Model characteristics

	Delta (1–3 Hz)	Theta (4–7 Hz)	Alpha (8–12 Hz)	Low beta (13–19 Hz)	High beta (20–30 Hz)
Lasso regression model					
Number of significant leads	1	1	5	3	6
Regression coefficients (min, max)	34.3	−15.0	−8.1, 22.4	−42.8, −2.2	−20.9, 57.7
Elastic net model					
Number of significant leads	4	5	11	9	20
Regression coefficients (min, max)	−1.0, 18.6	−30.9, 8.2	−3.5, 8.6	−17.5, −0.8	−11.0, 35.4

### 3.3 Corticospinal tract injury and baseline motor status

CST overlap injury (*R* ^2^ = 6.2%, *p* = .21) did not predict a significant percentage of variance in motor recovery. Despite collinearity likely existing between baseline motor status (UEFM) and motor recovery (change in FIM‐motor), we also examined the amount of variance in motor recovery explained by baseline motor status and found it to be nonsignificant (*R* ^2^ = 1.1%, *p* = .54).

As an additional secondary analysis, we examined if EEG coherence contains distinct information about motor recovery apart from CST injury and baseline motor status. We repeated lasso regression procedures using iM1 EEG coherence to predict the raw residuals acquired from the linear regression models of motor recovery involving CST injury and baseline motor status. EEG coherence with iM1 across a 1–30 Hz band predicted 54.5% and 58.6% of the variance in error from motor recovery predictive models with CST overlap injury and baseline motor status predictors, respectively, suggesting that EEG coherence, CST injury, and a clinical assessment of baseline motor status contain distinct information about motor recovery.

### 3.4 Evolution of coherence measurements during IRF stay

A subset of individuals completed additional EEG recordings during their IRF stay at 1 week (visit 2, *n* = 18) and 2 weeks (visit 3, *n* = 8) following their initial EEG recording. To better understand how key findings identified by lasso and elastic net models evolved over time since the initial visit at IRF admission (baseline), we examined these serial recordings. We focused on the findings from the EEG predictor model (Figure 2), that is, high beta iM1 coherence with leads overlying SMA and ipsilesional parietal (iPAR) and ventral premotor (iPMv) regions.

High beta iM1 coherence with SMA demonstrated slight increases from baseline to visit 2 in 11 of the 18 participants (median = 0.03, range = −0.12 to 0.15) and also from baseline to visit 3 in 6 of 8 participants (median = 0.06, range = −0.03 to 0.21). Similar findings were observed for high beta iM1 coherence with iPAR. From baseline to visit 2, 8 of the 18 participants showed increases in coherence (median = −0.01, range = −0.27 to 0.17), and from baseline to visit 3 with 4 of the 8 participants demonstrating increases (median = −0.008, range = −0.16 to 0.12). Lastly, 9 of 18 participants demonstrated slight to moderate increases in high beta iM1 coherence with iPMv from baseline to visit 2 (median = −0.004, range = −0.44 to 0.50) and 4 of the 8 participants from baseline to visit 3 (median = 0.003, range = −0.33 to 0.16). Table 3 provides additional information from serial EEG recordings. Reductions in beta iM1 coherence with iPMv from baseline to visit 3 trended toward greater motor recovery (increased FIM‐motor scores) during this time (*r* = 0.64, *p* = .06).

**TABLE 3:** Serial coherence measurements

High beta (20–30 Hz) coherence measure	Baseline (*n* = 27)	Visit 2 (*n* = 18)	Visit 3 (*n* = 8)
iM1‐SMA	0.19 ± 0.11	0.21 ± 0.09	0.23 ± 0.10*
iM1‐iPAR	0.18 ± 0.08	0.17 ± 0.09	0.14 ± 0.06
iM1‐iPMv	0.50 ± 0.23	0.52 ± 0.23	0.52 ± 0.17

*Note*: Values presented as mean ± SD.

Abbreviations: iM1, ipsilesional primary motor cortex; iPAR, ipsilesional parietal cortex; iPMv, ipsilesional ventral premotor cortex.

* Significant increase from baseline (*t* = 2.11, *p* = .04).

## 4 DISCUSSION

Applying regularization techniques to feature‐rich dense‐array EEG strongly predicted motor recovery during early stroke inpatient hospitalization. A cross‐validated lasso regression approach found that EEG coherence between iM1 and 16 leads across a 1–30 Hz frequency band explained over 60% of the variance in motor recovery in our cohort of individuals residing in an IRF. Adjusting the regression penalty consistent with an elastic net regression approach revealed the broader set of leads responsible for predictive generalizability at the small cost of predictive power. Together, these findings emphasize the relevance of functional neural connections in poststroke motor recovery prediction.

Hypothesis‐driven predictive models, conceptualized from our prior work (Cassidy et al., 2020; Wu et al., 2016), emphasize delta and beta oscillatory contributions from leads overlying bilateral primary and secondary motor regions. Our data‐driven model of motor recovery prediction also emphasizes beta oscillations but now also acknowledges alpha oscillations. In contrast to our hypothesis‐driven model, leads overlying nonmotor regions were acknowledged and lower frequency bands (delta and theta) provided only minor influence on motor recovery. We discuss these findings and others in greater detail below.

### 4.1 Coherence of oscillations predicts motor recovery

An important finding was that EEG signal coherence, not power, predicted short‐term motor recovery in early stroke rehabilitation. There are limited data published to date using brain mapping studies to identify response to rehabilitation therapy received in an IRF setting after stroke despite the fact that billions of dollars are spent each year in the United States and that IRFs are an intense rehabilitation environment (Winstein et al., 2016) provided to approximately 25% of patients with stroke (Benjamin et al., 2017).

Pascal Fries's “communication‐through‐coherence” hypothesis (Fries, 2005) asserts that effective neuronal communication between two neuronal groups depends on the coherence of neural oscillations between them. Initially intended to account for potential mechanisms underlying cognitive dynamics and flexibility, this notion of coherence driving communication extrapolates to several clinical disease states, including stroke, whereby disordered neural connectivity and, hence, communication, contribute to the pathology and functional behavioral deficits of the disease. Resting‐state fMRI work in stroke has shown disruptions in neural connectivity and cross‐sectional associations with motor status (Carter et al., 2010) and longitudinal associations with motor recovery (Park et al., 2011). The current work joins a growing body of EEG literature in stroke depicting similar associations between connectivity and motor behavior (Dubovik et al., 2013; Hoshino, Oguchi, Inoue, Hoshino, & Hoshiyama, 2020; Nicolo et al., 2015; Wu et al., 2015) that emphasize the relevance of oscillatory‐mediated communication between cortical regions rather than simply the magnitude of a frequency‐specific signal. Two distinct features of the current work are the study of individuals days after their stroke, at the start of a period of extraordinary brain plasticity, and that we predicted motor *function*, rather than impairment, using coherence measures.

### 4.2 Multiple regions and oscillation frequencies contribute to motor recovery

The EEG leads identified through lasso and elastic net regression represent a mixture of frequency bands encompassing bilateral hemispheres overlying motor and nonmotor regions. Overall, higher (beta and alpha) frequency bands contributed most to the prediction model, particularly high beta leads overlying SMA, iPAR, and iPMv. The model also revealed a few sparse leads expressing lower (delta and theta) frequencies (Table 2). Elastic net regression identified additional high beta frequency band leads overlying contralesional frontal, parietal, and temporal–parietal regions along with SMA along with additional alpha leads overlying contralesional temporal–parietal and iPAR regions. Contributions from these particular frequency bands are discussed in greater detail below.

The inclusion of leads overlying nonmotor regions may reflect more generalized brain activity characteristic of a resting brain state as compared to brain activity in task or event‐related EEG paradigms probing specific functional domains (Quandt et al., 2019). However, one of the first studies to predict long‐term poststroke recovery using fMRI activity (task‐based) acquired early following stroke refutes this argument by reporting a spatially diffuse pattern of brain activity associated with recovery (Marshall et al., 2009). These findings parallel earlier task‐oriented fMRI studies depicting brain activation in areas other than primary motor cortex involving cingulate, temporal, and striate cortices, for example (Ward, Brown, Thompson, & Frackowiak, 2003b). Further supporting these findings and ours is an increased appreciation that input from nonmotor brain regions and networks influence motor system function in healthy participants and plasticity after stroke (Egger et al., 2021; Lin et al., 2021). Our finding of EEG leads overlying nonmotor regions in bilateral hemispheres may reflect one of many ongoing injury‐ or recovery‐related processes, such as compensatory neuroplasticity mechanisms (Park et al., 2011; Ward, Brown, Thompson, & Frackowiak, 2003a), diaschisis (Fornito, Zalesky, & Breakspear, 2015), and contributions from widespread network modulation. Indeed, recent work has shown an upregulation of frontoparietal network connectivity following stroke (Bönstrup et al., 2018; Hordacre et al., 2021) and attentional‐control network influence on motor performance in stroke (Rinne et al., 2018).

Another notable finding of this work was the absence of universally positive or negative associations between coherence in a frequency‐specific band with motor recovery. Low beta iM1 coherence was the exception that demonstrated negative associations with motor recovery across all three leads identified by lasso and across all nine leads identified by elastic net (Table 2). The distribution of low beta leads overlying iPAR and contralesional temporal and SMA regions closely aligns with the distribution of many of the high beta leads negatively associated with motor recovery. These findings imply that beta coherence between iM1 and these regions in early stroke recovery is maladaptive.

Given the consistency of findings showing the attenuation of beta oscillations following neural injury (Dubovik et al., 2012; Foreman & Claassen, 2012; Wu et al., 2016) and treatment‐induced improvement in motor status associated with heightened beta activity (Pellegrino et al., 2012; Wu et al., 2015), one may conclude positive associations between enhanced M1 beta coherence and motor recovery. Nicolo et al. (2015) observed positive associations between motor function improvement at 3 months poststroke and baseline (2–3 weeks poststroke) beta coherence between ipsilesional motor areas with the rest of the cortex (Nicolo et al., 2015). Similarly, we identified positive associations between motor recovery and high beta iM1 connectivity with leads overlying iPAR (lasso model, Figure 2a) and contralesional frontal (elastic net model, Figure 2b) regions. However, negative associations with motor recovery also existed in this frequency band entailing leads overlying SMA (Figure 2). The coexistence of positive and negative correlations with motor recovery within the high beta frequency band recognizes that some forms of connectivity represent behaviorally favorable changes while others do not. The positive associations that we observed may support the expanding role of beta oscillations beyond sensorimotor function (Pfurtscheller & Da Silva, 1999) and a cortical idling state (Pfurtscheller, Stancak Jr., & Neuper, 1996). Beta frequency oscillations are now thought to mediate widespread cortical network communication (Bastos et al., 2015), top‐down information processing (Engel & Fries, 2010), and feedback communication (Bastos et al., 2015). Hence, beta coherence involving frontal and parietal regions may further highlight beta activity contributions to visual processing (Piantoni, Kline, & Eagleman, 2010), working memory (Siegel, Warden, & Miller, 2009), and response inhibition (Jha et al., 2015) which may prove relevant even in a resting‐state condition whereby participants receive instruction to minimize movement and fixate on a visual target.

Two possibilities, underscoring the conflicting role of SMA in motor recovery in the literature, may explain the negative association between motor recovery and high beta iM1 with leads overlying SMA. First, several neuroimaging studies in stroke have shown behaviorally relevant contributions from secondary motor regions, including SMA, to motor recovery (Cramer et al., 1997; Grefkes et al., 2008; Rehme, Eickhoff, Wang, Fink, & Grefkes, 2011) via cortico‐cortical connections with M1 (Luppino, Matelli, Camarda, & Rizzolatti, 1993). Akin with functional connectivity studies demonstrating reduced or abnormal connectivity patterns following stroke (Carter et al., 2010), effective connectivity work involving dynamic causal modeling of task‐induced fMRI signals have shown diminished coupling between ipsilesional SMA and M1 early after stroke that gradually increases over time with recovery (Grefkes et al., 2008; Rehme et al., 2011). Applied to our findings, the negative association between baseline coherence between leads overlying these regions with motor recovery might indicate a greater capacity for change in the form of enhanced coherence between these regions over time that parallels motor recovery. However, the inspection of serial EEG recordings revealed only slight increases in iM1‐SMA coherence over time that were not related to behavioral recovery. The second possible explanation follows work by Thibaut et al. (2017) that observed an “excess” of high beta activity in the ipsilesional hemisphere that negatively correlated with motor function (Thibaut et al., 2017) corresponding with seminal fMRI work showing negative associations between BOLD signal activity in SMA and other secondary motor regions with motor recovery (Ward et al., 2003a). Therefore, this finding may also reflect maladaptive forms of plasticity or an inefficient adaptive response to weakness following stroke. In our case, greater beta iM1 coherence with SMA corresponds to a poorer outlook.

Past work has also shown interactions between functional connectivity after stroke and white matter (CST injury) status (Carter et al., 2012; Guggisberg, Nicolo, Cohen, Schnider, & Buch, 2017; Quandt et al., 2019). For instance, Quandt et al. (2019) found that the magnitude of beta desynchronization in SMA during reaching and grasping activities was dependent on CST integrity after stroke (Quandt et al., 2019). The size of our current cohort limited additional examination of CST injury sub‐cohorts using lasso and elastic net models. Hence, future larger‐scale work should examine the role of ipsilesional beta coherence across various patterns of motor system injury, to refine our understanding of how such coherence is related to white matter injury and early poststroke motor recovery.

Over a span of 2 weeks following IRF admission, group averages from serial EEG recordings (Table 3) showed a significant increase in high beta iM1 coherence with leads overlying SMA and trends of decreasing and increasing high beta iM1 coherence with leads overlying iPAR and iPMV, respectively. The lack of significant associations between these coherence changes with motor recovery reinforces our statistical approach and resulting lasso model (Figure 2a) that illustrates contributions to motor recovery from leads representing multiple frequency bands encompassing a 1–30 Hz spectrum. Therefore, we surmise that *changes* in coherence occurring across a variety of frequencies concurrently best encapsulate motor recovery.

Relatedly, lasso and elastic net models also indicated the involvement of alpha coherence in early poststroke motor recovery (Figure 2), most apparent in leads overlying the contralesional temporal–parietal region that positively related to motor recovery. Our findings both align and contrast with previous work in patients with stroke (Dubovik et al., 2012; Westlake et al., 2012) that also demonstrated correlations between resting‐state alpha coherence and motor status (Dubovik et al., 2012) and recovery (Westlake et al., 2012). In these studies, however, alpha coherence involving contralesional motor regions demonstrated negative associations with motor status and recovery. Dubovik et al. (2012) compared the presence of contralesional alpha “hyper‐synchrony” to interhemispheric inhibition imbalances favoring the contralesional hemisphere (Murase, Duque, Mazzocchio, & Cohen, 2004). A key distinction between our work and these studies is the timeframe poststroke entailing a timescale of days, versus months to years poststroke (Dubovik et al., 2012; Westlake et al., 2012). Applied to our findings, the generation of alpha activity mediated by cortico‐cortical connections (da Silva, Vos, Mooibroek, & Van Rotterdam, 1980) in combination with alpha oscillation involvement in cognition and memory function (Klimesch, 1999) suggest that early poststroke utilization of these neural substrates or networks is advantageous to motor recovery. In support, Wu et al. (2011) found that individuals depicting higher interhemispheric synchrony of alpha activity at 7 days poststroke demonstrated better motor recovery outcomes than those with lower interhemispheric alpha frequency (Wu et al., 2011). Serial EEG recordings over a 6–12‐month timeframe may bring these various results together and thereby enhance our understanding of alpha coherence in both short‐ and long‐term motor recovery while clarifying to what extent intra‐ and interhemipsheric connections in the alpha frequency band modulate with time and recovery.

### 4.3 Electroencephalography power, corticospinal tract injury, and baseline motor status did not predict early motor recovery

EEG power across a 1–30 Hz spectrum did not predict motor recovery. This finding does not necessarily imply an absence of correlation between relative power and motor recovery but, rather, it suggests that there is no predictive combination of variables. A lack of prediction from EEG relative power contrasts with findings from previous studies depicting associations between poststroke outcomes and EEG power measurements including brain symmetry indices and power ratios of various frequencies (Chiarelli et al., 2020; Cuspineda et al., 2007; Sheorajpanday, Nagels, Weeren, van Putten, & De Deyn, 2011). A key feature between these studies, including a recent study applying machine learning to predict functional status (Chiarelli et al., 2020), and ours is that the former utilized global assessments of poststroke severity and disability such as the NIH Stroke Scale and modified Rankin Scale as a primary outcome measure. Combined with our findings, this work suggests that EEG power measurements are more useful in the prediction of global poststroke outcomes that capture a person's overall functional state, whereas connectivity measurements best encapsulate domain‐specific recovery such as motor recovery. Stroke is a complex condition, and a single type of biomarker is unlikely to prove useful for addressing all clinical questions, at all time points, across all types of behavioral outcome measures. Future work discerning which biomarkers are optimal predictors across various parameters, for example, time poststroke, impairment level, primary outcome measure, time window to predict, and so on, will mitigate these discrepancies while advancing the application of functional neuroimaging‐based biomarkers in stroke recovery.

Similarly, CST injury and baseline motor status also demonstrated poor performance in the prediction of motor recovery. These findings contrast past work that showed significant prediction of motor recovery from initial UEFM scores (Zarahn et al., 2011) and identical measurement of CST injury (Lin et al., 2019) acquired early poststroke. However, an important distinction between these studies and ours was the focus on motor recovery across the first 3 months poststroke. This study focused on recovery during IRF hospitalization that spanned an average of 3 weeks following admittance. Our findings suggest that CST injury and initial UEFM scores do not adequately predict relatively brief periods of recovery occurring early after stroke. While the prediction of 3‐ and 6‐month poststroke recovery outcomes has merit, the prediction of outcomes during inpatient hospitalization poststroke is also invaluable and necessitates further investigation given that this timeframe influences hospital discharge planning and shapes patient and caregiver recovery expectations. Our sample also demonstrated considerable variance in CST injury and baseline motor status (Table 1), which may have also affected prediction. Recent work demonstrating different motor recovery trajectories among individuals (van der Vliet et al., 2020) combined with previous work asserting that the predictive value of biomarkers varies among motor impairment subgroups (Stewart et al., 2017) further support this reasoning.

### 4.4 EEG coherence contains unique information about motor recovery

When we repeated lasso regression procedures using EEG coherence to predict the *raw residuals* from generalized linear regression models involving CST injury and baseline motor status as motor recovery predictors, we found that EEG coherence still explained over 50% of the variance. This finding supports the overall conclusion that functional neuroimaging contains distinct information about motor recovery apart from structural neuroimaging and clinical assessments and is concordant with past work demonstrating better predictive performance from multivariate models containing both structural and functional neuroimaging measurements (Quinlan et al., 2015; Stinear, Barber, Petoe, Anwar, & Byblow, 2012) than models containing either measure alone. Recent work has shown interactions between structural and functional connectivity measurements (Hordacre et al., 2021; Quandt et al., 2019) and clinical recovery status (Guggisberg et al., 2017) which encourages future work to examine structure, function, and clinical status in concert with one another.

### 4.5 Strengths and limitations

This study presents a number of important strengths and limitations. The cohort featured in this study encompassed individuals residing in a therapeutically rich environment early after their stroke when the potential for motor (re)learning through neuroplasticity is most profound. Mechanisms of plasticity in this setting have not been widely examined. Utilization of dense‐array EEG over multiple frequency bands facilitated a data‐driven approach to motor recovery prediction during early stroke rehabilitation that highlighted several relevant functional connections with iM1. Serial EEG recordings acquired during inpatient rehabilitation hospitalization provided a preliminary account of how some of these key functional connections evolve over time. While prediction of long‐term recovery outcomes has merit, this study's focus on motor recovery during initial rehabilitation hospitalization, specifically in an IRF setting where individuals spend an average of 2–3 weeks, specifically warrants additional investigation. Admission to an IRF is determined by medical personnel based on their clinical judgment of an individual's ability to successfully participate in several hours of therapy per day, and our findings support bedside brain mapping over bedside clinical assessments at identifying clinical assessment recovery potential. For a data‐driven study focused on motor recovery prediction poststroke, we acknowledge the small sample size, which also limited further examination of specific CST injury and impairment in sub‐cohorts. Therefore, further studies are necessary to validate the present findings.

Our coherence analyses focused on whole brain connectivity with iM1 since this region is a prime therapeutic target and key region for the restitution of motor function after stroke. By limiting our seed region to iM1, we acknowledge that several functional connections potentially relevant to motor recovery were not considered in the model. This work is an initial step in motor recovery prediction using EEG coherence in a data‐driven approach. Future work may therefore examine multiple seed regions across various cohorts to advance our initial findings and interpretation of findings. We emphasize caution when interpreting the performance of both lasso and elastic net models. It is important to mention that lasso poses inherent limitations related to the selection of features and degenerate solution space. Relatedly, the model derived from elastic net regression, utilized in this study for the purpose of interpretation vs. prediction, may have overfit the data with the inclusion of additional variables, that is, EEG leads. Our non‐nested cross‐validation framework may have also inflated the performance of our models. Lastly, spatial resolution and volume conduction are obstacles frequently encountered in EEG studies. Dense‐array EEG systems partially mitigate spatial resolution issues; however, this work did not employ additional strategies such as source localization or spatial filtering since these techniques may further distort the data (Nunez et al., 2019).

## 5 CONCLUSIONS

The heterogeneity of stroke propelled the development of neuroimaging‐based biomarkers to enhance the prediction of clinical and treatment outcomes. Biomarkers that capture functional brain activity, particularly neural oscillations, may provide valuable information related to stroke rehabilitation and may further compliment information provided by more established structural biomarkers such as CST injury. Utilizing a data‐driven approach with lasso regression, this study investigated the predictive performance of EEG‐derived neural oscillations during early stroke rehabilitation. Our main finding was that EEG coherence between leads overlying iM1 (seed region) and 16 leads overlying ipsi‐ and contralesional hemispheres explained nearly 62% of the variance in motor recovery during IRF hospitalization outperforming EEG power, baseline motor status, and CST injury. The coherence of neural oscillations, based on the consistency of phase and amplitude differences across time, reflects communication between spatially distinct neural populations (Fries, 2005) and, thus, is an important factor in early poststroke motor recovery.

The inclusion of leads representing frequencies across a 1–30 Hz spectrum together with their spatial distribution spanning bilateral hemispheres demonstrated no frequency or location‐dependent direction of association with motor recovery. This finding illustrates the complex nature of motor recovery and the mechanisms of neuroplasticity mediating this process. Furthermore, model contributions from leads representing various frequency bands and scalp locations support the concept of motor recovery as the product of multiple interactions across various nonmotor neural networks. In comparison to traditional hypothesis‐based prediction models, data‐driven models may best capture these network interactions. This work identified several functional connections in specific frequency bands that encourage further investigation to establish a more complete understanding of circuit and network level activity in support of early poststroke motor recovery and the utility of EEG‐acquired oscillations as motor recovery biomarkers in stroke rehabilitation.

## CONFLICT OF INTEREST

Dr. Cramer consults for Abbvie, Constant Therapeutics, MicroTransponder, Neurolutions, SanBio, NeuExcell, Elevian, Medtronic, and TRCare. The other authors have no conflicts to report.

## Data Availability

The data that support the findings of this study are available on request from the corresponding author.

## References

[hbm25643-bib-0001] Bastos, A. M. , Vezoli, J. , Bosman, C. A. , Schoffelen, J.‐M. , Oostenveld, R. , Dowdall, J. R. , … Fries, P. (2015). Visual areas exert feedforward and feedback influences through distinct frequency channels. Neuron, 85(2), 390–401. 25556836 10.1016/j.neuron.2014.12.018

[hbm25643-bib-0002] Benjamin, E. J. , Blaha, M. J. , Chiuve, S. E. , Cushman, M. , Das, S. R. , Deo, R. , … Muntner, P. (2017). Heart disease and stroke Statistics‐2017 update: A report from the American Heart Association. Circulation, 135, e146–e603. 10.1161/cir.0000000000000485 28122885 PMC5408160

[hbm25643-bib-0003] Bönstrup, M. , Schulz, R. , Schön, G. , Cheng, B. , Feldheim, J. , Thomalla, G. , & Gerloff, C. (2018). Parietofrontal network upregulation after motor stroke. Neuroimage: Clinical, 18, 720–729. 29876261 10.1016/j.nicl.2018.03.006 PMC5987870

[hbm25643-bib-0004] Boyd, L. A. , Hayward, K. S. , Ward, N. S. , Stinear, C. M. , Rosso, C. , Fisher, R. J. , … Cramer, S. C. (2017). Biomarkers of stroke recovery: Consensus‐based core recommendations from the stroke recovery and rehabilitation roundtable. International Journal of Stroke, 12(5), 480–493. 10.1177/1747493017714176 28697711 PMC6791523

[hbm25643-bib-0005] Bunea, F. , She, Y. , Ombao, H. , Gongvatana, A. , Devlin, K. , & Cohen, R. (2011). Penalized least squares regression methods and applications to neuroimaging. NeuroImage, 55(4), 1519–1527. 21167288 10.1016/j.neuroimage.2010.12.028 PMC5485905

[hbm25643-bib-0006] Burke, E. , Dodakian, L. , See, J. , McKenzie, A. , Riley, J. D. , Le, V. , & Cramer, S. C. (2014). A multimodal approach to understanding motor impairment and disability after stroke. Journal of Neurology, 261(6), 1178–1186. 10.1007/s00415-014-7341-8 24728337

[hbm25643-bib-0007] Carter, A. R. , Astafiev, S. V. , Lang, C. E. , Connor, L. T. , Rengachary, J. , Strube, M. J. , … Corbetta, M. (2010). Resting interhemispheric functional magnetic resonance imaging connectivity predicts performance after stroke. Annals of Neurology, 67(3), 365–375. 10.1002/ana.21905 20373348 PMC2927671

[hbm25643-bib-0008] Carter, A. R. , Patel, K. R. , Astafiev, S. V. , Snyder, A. Z. , Rengachary, J. , Strube, M. J. , … Corbetta, M. (2012). Upstream dysfunction of somatomotor functional connectivity after corticospinal damage in stroke. Neurorehabilitation and Neural Repair, 26(1), 7–19. 10.1177/1545968311411054 21803932 PMC3822763

[hbm25643-bib-0009] Cassidy, J. M. , Wodeyar, A. , Wu, J. , Kaur, K. , Masuda, A. K. , Srinivasan, R. , & Cramer, S. C. (2020). Low‐frequency oscillations are a biomarker of injury and recovery after stroke. Stroke, 51(5), 1442–1450. 32299324 10.1161/STROKEAHA.120.028932 PMC7188582

[hbm25643-bib-0010] Chiarelli, A. M. , Croce, P. , Assenza, G. , Merla, A. , Granata, G. , Giannantoni, N. M. , … Zappasodi, F. (2020). Electroencephalography‐derived prognosis of functional recovery in acute stroke through machine learning approaches. International Journal of Neural Systems, 30, 2050067. 33236654 10.1142/S0129065720500677

[hbm25643-bib-0011] Cole, J. H. (2020). Multi‐modality neuroimaging brain‐age in UK biobank: Relationship to biomedical, lifestyle and cognitive factors. Neurobiology of Aging., 92, 34–42. 32380363 10.1016/j.neurobiolaging.2020.03.014 PMC7280786

[hbm25643-bib-0012] Cramer, S. C. , Nelles, G. , Benson, R. R. , Kaplan, J. D. , Parker, R. A. , Kwong, K. K. , … Rosen, B. R. (1997). A functional MRI study of subjects recovered from hemiparetic stroke. Stroke, 28(12), 2518–2527. 9412643 10.1161/01.str.28.12.2518

[hbm25643-bib-0013] Cuspineda, E. , Machado, C. , Galan, L. , Aubert, E. , Alvarez, M. A. , Llopis, F. , … Avila, Y. (2007). QEEG prognostic value in acute stroke. Clinical EEG and Neuroscience, 38(3), 155–160. 17844945 10.1177/155005940703800312

[hbm25643-bib-0014] da Silva, F. L. (1991). Neural mechanisms underlying brain waves: From neural membranes to networks. Electroencephalography and Clinical Neurophysiology, 79(2), 81–93. 1713832 10.1016/0013-4694(91)90044-5

[hbm25643-bib-0015] da Silva, F. L. , Vos, J. , Mooibroek, J. , & Van Rotterdam, A. (1980). Relative contributions of intracortical and thalamo‐cortical processes in the generation of alpha rhythms, revealed by partial coherence analysis. Electroencephalography and Clinical Neurophysiology, 50(5–6), 449–456. 6160987 10.1016/0013-4694(80)90011-5

[hbm25643-bib-0016] Delorme, A. , & Makeig, S. (2004). EEGLAB: An open source toolbox for analysis of single‐trial EEG dynamics including independent component analysis. Journal of Neuroscience Methods, 134(1), 9–21. 10.1016/j.jneumeth.2003.10.009 15102499

[hbm25643-bib-0017] Dubovik, S. , Pignat, J. M. , Ptak, R. , Aboulafia, T. , Allet, L. , Gillabert, N. , … Guggisberg, A. G. (2012). The behavioral significance of coherent resting‐state oscillations after stroke. NeuroImage, 61(1), 249–257. 10.1016/j.neuroimage.2012.03.024 22440653

[hbm25643-bib-0018] Dubovik, S. , Ptak, R. , Aboulafia, T. , Magnin, C. , Gillabert, N. , Allet, L. , … Guggisberg, A. G. (2013). EEG alpha band synchrony predicts cognitive and motor performance in patients with ischemic stroke. Behavioural Neurology, 26(3), 187–189. 22713421 10.3233/BEN-2012-129007 PMC5214220

[hbm25643-bib-0019] Egger, P. , Evangelista, G. G. , Koch, P. J. , Park, C. H. , Levin‐Gleba, L. , Girard, G. , … Hummel, F. C. (2021). Disconnectomics of the rich club impacts motor recovery after stroke. Stroke, 52, 2115–2124. 10.1161/strokeaha.120.031541 33902299

[hbm25643-bib-0020] Engel, A. K. , & Fries, P. (2010). Beta‐band oscillations—Signalling the status quo? Current Opinion in Neurobiology, 20(2), 156–165. 10.1016/j.conb.2010.02.015 20359884

[hbm25643-bib-0021] Erani, F. , Zolotova, N. , Vanderschelden, B. , Khoshab, N. , Sarian, H. , Nazarzai, L. , … Yu, W. (2020). Electroencephalography might improve diagnosis of acute stroke and large vessel occlusion. Stroke, 51(11), 3361–3365. 32942967 10.1161/STROKEAHA.120.030150 PMC7606743

[hbm25643-bib-0022] Feigin, V. L. , Vos, T. , Alahdab, F. , Amit, A. M. L. , Bärnighausen, T. W. , Beghi, E. , … Desai, R. (2021). Burden of neurological disorders across the US from 1990–2017: A global burden of disease study. JAMA Neurology, 78(2), 165–176. 33136137 10.1001/jamaneurol.2020.4152 PMC7607495

[hbm25643-bib-0023] Foreman, B. , & Claassen, J. (2012). Quantitative EEG for the detection of brain ischemia. Critical Care, 16(2), 216. 10.1186/cc11230 22429809 PMC3681361

[hbm25643-bib-0024] Fornito, A. , Zalesky, A. , & Breakspear, M. (2015). The connectomics of brain disorders. Nature Reviews Neuroscience, 16(3), 159–172. 10.1038/nrn3901 25697159

[hbm25643-bib-0025] Fries, P. (2005). A mechanism for cognitive dynamics: Neuronal communication through neuronal coherence. Trends in Cognitive Sciences, 9(10), 474–480. 16150631 10.1016/j.tics.2005.08.011

[hbm25643-bib-0026] Grefkes, C. , Nowak, D. A. , Eickhoff, S. B. , Dafotakis, M. , Kust, J. , Karbe, H. , & Fink, G. R. (2008). Cortical connectivity after subcortical stroke assessed with functional magnetic resonance imaging. Annals of Neurology, 63(2), 236–246. 10.1002/ana.21228 17896791

[hbm25643-bib-0027] Guggisberg, A. G. , Nicolo, P. , Cohen, L. G. , Schnider, A. , & Buch, E. R. (2017). Longitudinal structural and functional differences between proportional and poor motor recovery after stroke. Neurorehabilitation and Neural Repair, 31(12), 1029–1041. 10.1177/1545968317740634 29130824 PMC6368856

[hbm25643-bib-0028] Günseli, E. , Fahrenfort, J. J. , van Moorselaar, D. , Daoultzis, K. C. , Meeter, M. , & Olivers, C. N. (2019). EEG dynamics reveal a dissociation between storage and selective attention within working memory. Scientific Reports, 9(1), 1–13. 31534150 10.1038/s41598-019-49577-0 PMC6751203

[hbm25643-bib-0029] Hoerl, A. E. , & Kennard, R. W. (1970). Ridge regression: Biased estimation for nonorthogonal problems. Technometrics, 12(1), 55–67.

[hbm25643-bib-0030] Hordacre, B. , Lotze, M. , Jenkinson, M. , Lazari, A. , Barras, C. D. , Boyd, L. , & Hillier, S. (2021). Fronto‐parietal involvement in chronic stroke motor performance when Corticospinal tract integrity is compromised. Neuroimage: Clinical, 29, 102558. 33513561 10.1016/j.nicl.2021.102558 PMC7841401

[hbm25643-bib-0031] Hoshino, T. , Oguchi, K. , Inoue, K. , Hoshino, A. , & Hoshiyama, M. (2020). Relationship between upper limb function and functional neural connectivity among motor related‐areas during recovery stage after stroke. Topics in Stroke Rehabilitation, 27(1), 57–66. 31535592 10.1080/10749357.2019.1658429

[hbm25643-bib-0032] Hua, K. , Zhang, J. , Wakana, S. , Jiang, H. , Li, X. , Reich, D. S. , … Mori, S. (2008). Tract probability maps in stereotaxic spaces: Analyses of white matter anatomy and tract‐specific quantification. NeuroImage, 39(1), 336–347. 17931890 10.1016/j.neuroimage.2007.07.053 PMC2724595

[hbm25643-bib-0033] Jensen, O. , Kaiser, J. , & Lachaux, J.‐P. (2007). Human gamma‐frequency oscillations associated with attention and memory. Trends in Neurosciences, 30(7), 317–324. 17499860 10.1016/j.tins.2007.05.001

[hbm25643-bib-0034] Jha, A. , Nachev, P. , Barnes, G. , Husain, M. , Brown, P. , & Litvak, V. (2015). The frontal control of stopping. Cerebral Cortex, 25(11), 4392–4406. 25754518 10.1093/cercor/bhv027 PMC4813761

[hbm25643-bib-0035] Klimesch, W. (1999). EEG alpha and theta oscillations reflect cognitive and memory performance: A review and analysis. Brain Research Reviews, 29(2–3), 169–195. 10209231 10.1016/s0165-0173(98)00056-3

[hbm25643-bib-0036] Knyazev, G. G. (2012). EEG delta oscillations as a correlate of basic homeostatic and motivational processes. Neuroscience and Biobehavioral Reviews, 36(1), 677–695. 10.1016/j.neubiorev.2011.10.002 22020231

[hbm25643-bib-0037] Kohannim, O. , Hibar, D. P. , Stein, J. L. , Jahanshad, N. , Hua, X. , Rajagopalan, P. , … De Zubicaray, G. I. (2012). Discovery and replication of gene influences on brain structure using LASSO regression. Frontiers in Neuroscience, 6, 115. 22888310 10.3389/fnins.2012.00115 PMC3412288

[hbm25643-bib-0038] Lin, D. J. , Cloutier, A. M. , Erler, K. S. , Cassidy, J. M. , Snider, S. B. , Ranford, J. , … Schwamm, L. H. (2019). Corticospinal tract injury estimated from acute stroke imaging predicts upper extremity motor recovery after stroke. Stroke, 50(12), 3569–3577. 31648631 10.1161/STROKEAHA.119.025898 PMC6878199

[hbm25643-bib-0039] Lin, D. J. , Erler, K. S. , Snider, S. B. , Bonkhoff, A. K. , DiCarlo, J. A. , Lam, N. , … Cramer, S. C. (2021). Cognitive demands influence upper extremity motor performance during recovery from acute stroke. Neurology, 96(21), e2576–e2586. 10.1212/wnl.0000000000011992 33858997 PMC8205451

[hbm25643-bib-0040] Luppino, G. , Matelli, M. , Camarda, R. , & Rizzolatti, G. (1993). Corticocortical connections of area F3 (SMA‐proper) and area F6 (pre‐SMA) in the macaque monkey. Journal of Comparative Neurology, 338(1), 114–140. 10.1002/cne.903380109 7507940

[hbm25643-bib-0041] Marshall, R. S. , Zarahn, E. , Alon, L. , Minzer, B. , Lazar, R. M. , & Krakauer, J. W. (2009). Early imaging correlates of subsequent motor recovery after stroke. Annals of Neurology, 65(5), 596–602. 19479972 10.1002/ana.21636 PMC2727702

[hbm25643-bib-0042] Meder, D. , & Siebner, H. R. (2018). Spectral signatures of neurodegenerative diseases: How to decipher them? Brain, 141(8), 2241–2244. 10.1093/brain/awy195 30060021

[hbm25643-bib-0043] Murase, N. , Duque, J. , Mazzocchio, R. , & Cohen, L. (2004). Influence of interhemispheric interactions on motor function in chronic stroke. Annals of Neurology, 55(3), 400–409. 14991818 10.1002/ana.10848

[hbm25643-bib-0044] Nicolo, P. , Rizk, S. , Magnin, C. , Pietro, M. D. , Schnider, A. , & Guggisberg, A. G. (2015). Coherent neural oscillations predict future motor and language improvement after stroke. Brain, 138(Pt 10), 3048–3060. 10.1093/brain/awv200 26163304

[hbm25643-bib-0045] Nunez, P. L. , Nunez, M. D. , & Srinivasan, R. (2019). Multi‐scale neural sources of EEG: Genuine, equivalent, and representative. A tutorial review. Brain Topography, 32(2), 193–214. 10.1007/s10548-019-00701-3 30684161

[hbm25643-bib-0046] Park, C. H. , Chang, W. H. , Ohn, S. H. , Kim, S. T. , Bang, O. Y. , Pascual‐Leone, A. , & Kim, Y. H. (2011). Longitudinal changes of resting‐state functional connectivity during motor recovery after stroke. Stroke, 42(5), 1357–1362. 10.1161/strokeaha.110.596155 21441147 PMC3589816

[hbm25643-bib-0047] Pellegrino, G. , Tomasevic, L. , Tombini, M. , Assenza, G. , Bravi, M. , Sterzi, S. , … Cavallo, G. (2012). Inter‐hemispheric coupling changes associate with motor improvements after robotic stroke rehabilitation. Restorative Neurology and Neuroscience, 30(6), 497–510. 22868224 10.3233/RNN-2012-120227

[hbm25643-bib-0048] Pfurtscheller, G. , Brunner, C. , Schlögl, A. , & Da Silva, F. L. (2006). Mu rhythm (de) synchronization and EEG single‐trial classification of different motor imagery tasks. NeuroImage, 31(1), 153–159. 16443377 10.1016/j.neuroimage.2005.12.003

[hbm25643-bib-0049] Pfurtscheller, G. , & Da Silva, F. L. (1999). Event‐related EEG/MEG synchronization and desynchronization: Basic principles. Clinical Neurophysiology, 110(11), 1842–1857. 10576479 10.1016/s1388-2457(99)00141-8

[hbm25643-bib-0050] Pfurtscheller, G. , Stancak, A., Jr. , & Neuper, C. (1996). Post‐movement beta synchronization. A correlate of an idling motor area? Electroencephalography and Clinical Neurophysiology, 98(4), 281–293. 8641150 10.1016/0013-4694(95)00258-8

[hbm25643-bib-0051] Piantoni, G. , Kline, K. A. , & Eagleman, D. M. (2010). Beta oscillations correlate with the probability of perceiving rivalrous visual stimuli. Journal of Vision, 10(13), 18–18. 10.1167/10.13.18 21149311

[hbm25643-bib-0052] Quandt, F. , Bönstrup, M. , Schulz, R. , Timmermann, J. E. , Mund, M. , Wessel, M. J. , & Hummel, F. C. (2019). The functional role of beta‐oscillations in the supplementary motor area during reaching and grasping after stroke: A question of structural damage to the corticospinal tract. Human Brain Mapping, 40(10), 3091–3101. 30927325 10.1002/hbm.24582 PMC6865486

[hbm25643-bib-0053] Quinlan, E. B. , Dodakian, L. , See, J. , McKenzie, A. , Le, V. , Wojnowicz, M. , … Cramer, S. C. (2015). Neural function, injury, and stroke subtype predict treatment gains after stroke. Annals of Neurology, 77(1), 132–145. 25382315 10.1002/ana.24309 PMC4293339

[hbm25643-bib-0054] Rehme, A. K. , Eickhoff, S. B. , Wang, L. E. , Fink, G. R. , & Grefkes, C. (2011). Dynamic causal modeling of cortical activity from the acute to the chronic stage after stroke. NeuroImage, 55(3), 1147–1158. 21238594 10.1016/j.neuroimage.2011.01.014 PMC8053821

[hbm25643-bib-0055] Rinne, P. , Hassan, M. , Fernandes, C. , Han, E. , Hennessy, E. , Waldman, A. , … Bentley, P. (2018). Motor dexterity and strength depend upon integrity of the attention‐control system. Proceedings of the National Academy of Sciences of the United States of America, 115(3), E536–e545. 10.1073/pnas.1715617115 29284747 PMC5776987

[hbm25643-bib-0056] Saes, M. , Meskers, C. G. , Daffertshofer, A. , van Wegen, E. E. , & Kwakkel, G. (2020). Are early measured resting‐state EEG parameters predictive for upper limb motor impairment six months poststroke? Clinical Neurophysiology, 132(1), 56–62. 33248434 10.1016/j.clinph.2020.09.031

[hbm25643-bib-0057] Sheorajpanday, R. V. , Nagels, G. , Weeren, A. J. , van Putten, M. J. , & De Deyn, P. P. (2011). Quantitative EEG in ischemic stroke: Correlation with functional status after 6 months. Clinical Neurophysiology, 122(5), 874–883. 20961806 10.1016/j.clinph.2010.07.028

[hbm25643-bib-0058] Siegel, M. , Warden, M. R. , & Miller, E. K. (2009). Phase‐dependent neuronal coding of objects in short‐term memory. Proceedings of the National Academy of Sciences, 106(50), 21341–21346. 10.1073/pnas.0908193106 PMC2779828 19926847

[hbm25643-bib-0059] Srinivasan, R. , Winter, W. R. , Ding, J. , & Nunez, P. L. (2007). EEG and MEG coherence: Measures of functional connectivity at distinct spatial scales of neocortical dynamics. Journal of Neuroscience Methods, 166(1), 41–52. 10.1016/j.jneumeth.2007.06.026 17698205 PMC2151962

[hbm25643-bib-0060] Stewart, J. C. , Dewanjee, P. , Tran, G. , Quinlan, E. B. , Dodakian, L. , McKenzie, A. , … Cramer, S. C. (2017). Role of corpus callosum integrity in arm function differs based on motor severity after stroke. Neuroimage: Clinical, 14, 641–647. 10.1016/j.nicl.2017.02.023 28348955 PMC5357692

[hbm25643-bib-0061] Stinear, C. M. , Barber, P. A. , Petoe, M. , Anwar, S. , & Byblow, W. D. (2012). The PREP algorithm predicts potential for upper limb recovery after stroke. Brain, 135(Pt 8), 2527–2535. 10.1093/brain/aws146 22689909

[hbm25643-bib-0062] Thibaut, A. , Simis, M. , Battistella, L. R. , Fanciullacci, C. , Bertolucci, F. , Huerta‐Gutierrez, R. , … Fregni, F. (2017). Using brain oscillations and Corticospinal excitability to understand and predict post‐stroke motor function. Frontiers in Neurology, 8, 187. 10.3389/fneur.2017.00187 28539912 PMC5423894

[hbm25643-bib-0063] Tibshirani, R. (1996). Regression shrinkage and selection via the lasso. Journal of the Royal Statistical Society: Series B (Methodological), 58(1), 267–288.

[hbm25643-bib-0064] Tomassini, A. , Ambrogioni, L. , Medendorp, W. P. , & Maris, E. (2017). Theta oscillations locked to intended actions rhythmically modulate perception. eLife, 6, e25618. 28686161 10.7554/eLife.25618 PMC5553936

[hbm25643-bib-0065] van der Vliet, R. , Selles, R. W. , Andrinopoulou, E. R. , Nijland, R. , Ribbers, G. M. , Frens, M. A. , … Kwakkel, G. (2020). Predicting upper limb motor impairment recovery after stroke: A mixture model. Annals of Neurology, 87(3), 383–393. 31925838 10.1002/ana.25679 PMC7065018

[hbm25643-bib-0066] Ward, N. S. , Brown, M. M. , Thompson, A. J. , & Frackowiak, R. S. (2003a). Neural correlates of motor recovery after stroke: A longitudinal fMRI study. Brain, 126(Pt 11), 2476–2496. 10.1093/brain/awg245 12937084 PMC3717457

[hbm25643-bib-0067] Ward, N. S. , Brown, M. M. , Thompson, A. J. , & Frackowiak, R. S. (2003b). Neural correlates of outcome after stroke: A cross‐sectional fMRI study. Brain, 126(Pt 6), 1430–1448. 10.1093/brain/awg145 12764063 PMC3717456

[hbm25643-bib-0068] Westlake, K. P. , Hinkley, L. B. , Bucci, M. , Guggisberg, A. G. , Byl, N. , Findlay, A. M. , … Nagarajan, S. S. (2012). Resting state alpha‐band functional connectivity and recovery after stroke. Experimental Neurology, 237(1), 160–169. 10.1016/j.expneurol.2012.06.020 22750324 PMC3646713

[hbm25643-bib-0069] Winstein, C. J. , Stein, J. , Arena, R. , Bates, B. , Cherney, L. R. , Cramer, S. C. , … Zorowitz, R. D. (2016). Guidelines for adult stroke rehabilitation and recovery: A guideline for healthcare professionals from the American Heart Association/American Stroke Association. Stroke, 47(6), e98–e169. 10.1161/str.0000000000000098 27145936

[hbm25643-bib-0070] Wu, J. , Quinlan, E. B. , Dodakian, L. , McKenzie, A. , Kathuria, N. , Zhou, R. J. , … Srinivasan, R. (2015). Connectivity measures are robust biomarkers of cortical function and plasticity after stroke. Brain, 138(8), 2359–2369. 26070983 10.1093/brain/awv156 PMC4840951

[hbm25643-bib-0071] Wu, J. , Srinivasan, R. , Quinlan, E. B. , Solodkin, A. , Small, S. L. , & Cramer, S. C. (2016). Utility of EEG measures of brain function in patients with acute stroke. Journal of Neurophysiology, 115(5), 2399–2405. 10.1152/jn.00978.2015 26936984 PMC4922461

[hbm25643-bib-0072] Wu, W. , Sun, J. , Jin, Z. , Guo, X. , Qiu, Y. , Zhu, Y. , & Tong, S. (2011). Impaired neuronal synchrony after focal ischemic stroke in elderly patients. Clinical Neurophysiology, 122(1), 21–26. 20591730 10.1016/j.clinph.2010.06.003

[hbm25643-bib-0073] Zarahn, E. , Alon, L. , Ryan, S. L. , Lazar, R. M. , Vry, M.‐S. , Weiller, C. , … Krakauer, J. W. (2011). Prediction of motor recovery using initial impairment and fMRI 48 h poststroke. Cerebral Cortex, 21(12), 2712–2721. 10.1093/cercor/bhr047 21527788 PMC3209795

[hbm25643-bib-0074] Zou, H. , & Hastie, T. (2005). Regularization and variable selection via the elastic net. Journal of the Royal Statistical Society: Series B (Statistical Methodology), 67(2), 301–320.

